# Adoption of mHealth Technologies by Community Health Workers to Improve the Use of Maternal Health Services in Sub-Saharan Africa: Protocol for a Mixed Method Systematic Review

**DOI:** 10.2196/44066

**Published:** 2023-05-04

**Authors:** Chiyembekezo Kachimanga, Titus H Divala, Johannes C F Ket, Alexandra V Kulinkina, Haules R Zaniku, Joia Murkherjee, Daniel Palazuelos, Ibukun-Oluwa Omolade Abejirinde, Thomas van den Akker

**Affiliations:** 1 Athena Institute Vrije Universiteit Amsterdam Amsterdam Netherlands; 2 Clinical Department Partners In Health Malawi Neno Malawi; 3 School of Global and Public Health Kamuzu University of Health Sciences Blantyre Malawi; 4 Swiss Center for International Health Swiss Tropical and Public Health Institute Basel Switzerland; 5 University of Basel Basel Switzerland; 6 Neno District Hospital Ministry of Health Neno Malawi; 7 Community Health Department Partners In Health Boston, MA United States; 8 Women's College Hospital Institute for Health System Solutions and Virtual Care Toronto, ON Canada; 9 Dalla Lana School of Public Health University of Toronto Toronto, ON Canada

**Keywords:** community health worker, maternal health, mobile health, sub-Saharan Africa, systematic review, review method, maternal, maternity, gynecology, mHealth, narrative synthesis, review method, antenatal, post natal, women's health, health care provider, health worker, health care worker

## Abstract

**Background:**

Studies have shown that mobile health technologies (mHealth) enhance the use of maternal health services. However, there is limited evidence of the impact of mHealth use by community health workers (CHWs) on the use of maternal health services in sub-Saharan Africa.

**Objective:**

This mixed method systematic review will explore the impact of mHealth use by CHWs on the use of the maternal health continuum of care (antenatal care, intrapartum care, and postnatal care [PNC]), as well as barriers and facilitators of mHealth use by CHWs when supporting maternal health services.

**Methods:**

We will include studies that report the impact of mHealth by CHWs on the use of antenatal care, facility-based births, and PNC visits in sub-Saharan Africa. We will search 6 databases (MEDLINE, CINAHL, Web of Science, Embase, Scopus, and Africa Index Medicus), with additional articles identified from Google Scholar and manual screening of references of the included studies. The included studies will not be limited by language or year of publication. After study selection, 2 independent reviewers will perform title and abstract screening, followed by full-text screening to identify the final papers to be included. Data extraction and risk-of-bias assessment will be performed using Covidence software by 2 independent reviewers. We will use a Mixed Methods Appraisal Tool to perform risk-of-bias assessments on all included studies. Finally, we will perform a narrative synthesis of the outcomes, integrating information about the effect of mHealth on maternal health use and barriers and facilitators of mHealth use. This protocol follows the PRISMA-P (Preferred Reporting Items for Systematic Reviews and Meta-Analyses Protocols) guidelines.

**Results:**

In September 2022, we conducted an initial search in the eligible databases. After removing duplicates, we identified 1111 studies that were eligible for the title and abstract screening. We will finalize the full-text assessment for eligibility, data extraction, assessment of methodological quality, and narrative synthesis by June 2023.

**Conclusions:**

This systematic review will present new and up-to-date evidence on the use of mHealth by CHWs along the pregnancy, childbirth, and PNC continuum of care. We anticipate the results will inform program implementation and policy by highlighting the potential impacts of mHealth and presenting contextual factors that should be addressed to ensure the success of the programs.

**Trial Registration:**

PROSPERO CRD42022346364; https://www.crd.york.ac.uk/prospero/display_record.php?RecordID=346364

**International Registered Report Identifier (IRRID):**

DERR1-10.2196/44066

## Introduction

### Background

Sub-Saharan Africa (SSA) continues to have the highest burden of maternal morbidity and mortality in the world [1]. About two-thirds of all maternal deaths occur in this region alone [2], despite containing only 15% of the global population [3]. Low use of antenatal care (ANC), unattended home births, and low use of postnatal care (PNC) have been identified as some of the biggest contributors to high maternal morbidity and mortality [4]. Consequently, policy makers and researchers in many SSA countries are beginning to realize the need for innovative ways of increasing access to and improving the quality of care along the pregnancy, childbirth, and postpartum continuum of care [5].

In SSA, mobile health (mHealth) technologies hold the potential to enhance use and quality of care during pregnancy, childbirth, and postpartum continuum of care, and thereby contribute to the reduction of maternal morbidity and mortality [6]. Currently, there is no universal definition of mHealth [7,8]. The World Health Organization defines mHealth as the use of mobile and wireless devices to support public health and medical practices [9]. When used appropriately, mHealth can be used to enhance use and quality of care. Additionally, mHealth can improve communication between providers and patients, and serve as a tool for education and mobilization [10]. Due to these anticipated benefits, there is a push to introduce mHealth to community health workers (CHWs) [11]. This is now possible in many SSA countries due to increased mobile and wireless device coverage and reduced cost of mHealth technologies [12,13].

CHWs act as a bridge between communities and health facilities by directly providing preventive health services, health promotion, and social mobilization among other home-based services [14,15]. Their role in the provision of health services is also vital as they help to address cultural barriers to accessing health services [16]. Finally, CHWs act as voices of social change by advocating for the health and social needs of the communities they serve [17]. If implemented well, these roles help improve the use of key health services, including maternal health services. Mobile health technologies may enhance the key roles of CHWs.

Despite the potential positive impact, the use of mHealth technologies is associated with challenges, especially in low- and middle-income countries. These include technological challenges, such as concerns with data security and privacy [18], multiple platforms of mHealth, some of which are not interoperable [19], poor network [13], lack of power [20], and high initial and maintenance costs [21]. Additional social factors also affect the use of mHealth including low literacy [22], insufficient training [23], cultural unacceptability [24,25], apps designed in languages other than the language of the users [24], and lack of integration into national health systems [10].

Recent reviews of the use of mHealth among CHWs have mainly focused on pilot projects and the mHealth implementation process and uptake [21,26]. Some reviews have shown that mHealth use by CHWs is linked to increased motivation, performance, and overall retention of CHWs [19,23]. Mobile health has supported the training of CHWs [27], improved patient adherence to scheduled appointments [28], and improved data collection, analysis, and use [29]. However, little is known about the effect of mHealth use by CHWs on the use of maternal health services in SSA.

### Study Objectives

Due to the relative novelty and potential challenges associated with implementation in SSA, the impact of mHealth on maternal health outcomes will depend on addressing barriers while building on facilitators to implementation. This review will explore the use of mHealth by CHWs in SSA along the pregnancy, childbirth, and postpartum continuum of care. Specifically, we will assess the evidence on the effect of mHealth on maternal health use, while exploring how programs identified barriers and facilitators that may impact the implementation of mHealth.

Review questions include the following:

1. Does the use of mHealth by CHWs increase utilization of maternal health services (ANC, facility-based births, and PNC) in SSA?
2. What are the facilitators and barriers of mHealth use by CHWs in programs designed to increase the utilization of maternal health services (ANC, facility-based births, and PNC) in SSA?

While the first review question will be answered by quantitative study designs, the second review question will be answered by both qualitative and quantitative study designs. Initially, we conducted a preliminary search of PROSPERO, MEDLINE, and JBI Evidence Synthesis, and no current or in-progress systematic reviews on the topic were identified.

## Methods

We have used the PRISMA-P (Preferred Reporting Items for Systematic Reviews and Meta-Analyses Protocols) guidelines to structure this protocol [30].

### Ethics Approval

Ethical approval is not required as the data collection will not directly involve any human subjects or identifiable patient data.

### Eligibility Criteria

#### Participants

In this review, participants include (1) pregnant women at any gestational age, (2) pregnant women during intrapartum care, and (3) women within 42 days after giving birth. We will include these women when using ANC, intrapartum care, and PNC. Women accessing these services and having coexisting conditions, for example eclampsia, will also be included.

#### Intervention

The use of mHealth by CHWs to improve maternal health use is the focus of the review. Mobile health devices will include portable and wireless devices such as cell phones, feature phones, tablets, portable media players, and GPS trackers, among others. We will classify the mHealth tools based on the 12 “signal functions” defined by Labrique et al [31] (Table 1).

**Table 1:** Common mobile health uses.

Number	Common mobile health uses
1	Client education and behavior change communication
2	Point of care diagnostics and sensors
3	Registries or vital events tracking
4	Data collection and reporting
5	Electronic health records
6	Clinical decision support
7	Provider-to-provider communication
8	Scheduling and work plan management
9	Provider capacity building
10	Human resource management
11	Supply chain management
12	Financial transactions and incentives

CHWs will be defined based on the World Health Organization definition, “health workers based in communities, who are either paid or volunteers, who are not professionals, and who have fewer than 2 years of training but at least some training” [32]. We developed the CHW search strategy based on commonly known names for CHWs in various contexts [33]. We will include CHWs who were working in mHealth programs designed to increase the use of maternal health services (as stand-alone programs or integrated with other clinical programs), and which are implemented in SSA as defined by the World Bank Group country classification [34].

#### Comparator

For interventional studies included in this review, the comparator will be CHW programs that are not using mHealth interventions.

#### Outcomes

Our outcomes will include the following:

1. Measures of the use of mHealth on the use of maternal health services along the continuum of care:
  ANC: total number of ANC visits, women who attended ANC in the first trimester, women who attended 4 or more ANC visits, and women with 8 or more ANC visits
  Facility-based births: women who gave birth at health facilities
  PNC: total number of PNC visits; women who attended PNC within 2 days, 3-7 days, and 8-42 days after giving births
  Other maternal and reproductive health outcomes or services such as abortions, caesarean sections, family planning, and neonatal and child health outcomes will be excluded.
2. Facilitators to mHealth use by the CHWs
3. Barriers to mHealth use by the CHWs

#### Study Designs

We will include the following study designs: (1) randomized controlled designs, (2) observational studies with competent counterfactual designs (eg, quasi-experimental studies), (3) nonexperimental quantitative studies, (4) qualitative studies, and (5) mixed methods studies. Protocols, case series or reports, abstracts and conference proceedings, commentaries, policy briefs and other policy documents, systematic reviews, and other summary-type articles will be excluded. Although systematic reviews and other summary-type articles will be excluded, we will check the references in these articles to identify additional papers.

### Information Sources and Search Strategy

We will search the following databases for potential studies: (1) Scopus, (2) MEDLINE, (3) CINAHL, (4) Web of Science, (5) Embase, and (6) Africa Index Medicus. Additional articles will be searched using Google Scholar and hand searching in references of all included studies. Study search will not be limited to a time or language. The sample component of search terms and search strategy is presented in Table 2. The search strategy was formulated by JCFK, who is a medical information specialist at Vrije Universiteit Amsterdam, the Netherlands.

**Table 2:** Sample search strategy (MEDLINE).

Title	Search strategy
Part 1: defining the study population	exp Pregnant Women/ or exp Pregnancy/ or Midwifery/ or Nurse Midwives/ or exp Postpartum Period/ or Maternal Health Services/ or exp Maternal-Child Health Services/ or exp Perinatal Care/ or exp Prenatal Care/ or exp Parturition/ or Reproductive Health Services/ or Reproductive Health/ or exp Labor, Obstetric/ or Maternal Death/ or (pregnan* or obstetr* or midwive* or midwife* or mid-wive* or mid-wife* or antenatal or postnatal or postpartum or post-natal or post-partum or gravid* or partur* or birth* or neonatal* or neo-natal* or lactat* or puerper* or labor or labour or term-birth or prenatal* or perinatal* or child-birth* or childbirth* or newborn* or (obstetr* adj5 deliver*) or breastfe* or breast-fe* or bottle-fe* or reproductive-health* or (maternal adj5 (health* or care* or welfare* or healthcare* or service* or death* or mortal*)) or reproductive-health* or reproductive-service* or pre-natal* or prenatal* or antenatal* or safe-motherhood* or safe-mother-hood or breech*).ti,ab,kf.
Part 2: defining intervention—mHealth^a^	exp Cell Phones/ or exp Computers, Handheld/ or exp Internet/ or exp Medical Informatics/ or exp Mobile Applications/ or exp Multimedia/ or exp Nursing informatics/ or exp Patient Portals/ or exp Public Health Informatics/ or exp Telemedicine/ or exp Telenursing/ or exp User-Computer Interface/ or (android or app or apps or cell-phone* or cellular-phone* or desktop* or desk-top* or digital-health or digital-diagnostic-device* or distance-consult* or distance-counsel* or distant-consult* or e-diagnos* or e-coach* or econsult* or e-consult* or ediagnos* or ehealth* or e-health* or 7frica7n* or facebook or face-book or feature-phone* or fixed-laptop* or game or games or gamification or gaming or global-positioning-system* or gps or health-app* or health-kiosk* or health-technolog* or interactive-voice-response* or internet* or ipad or ipads or iphone* or i-pad or i-pads or i-phone* or laptop* or lap-top* or mhapp* or mh-app* or mhealth* or m-health* or mobile-app* or mobile-health* or mobile-technolog* or mobile-device* or mobile-phone* or palm-top* or palmtop* or patient-portal* or pda or pdas or personal-digital-assistant* or personal-electronic-health-record* or personal-health-record* or phone-app* or portable-media-player* or radio-frequency-identification* or rfid* or remote-consult* or remote-counsel* or satellite-phone* or serious-gam* or smartphone* or smart-phone* or smart-phone* or sms or social-media* or tablets or tablet or tele-app* or tele-care or tele-consult* or tele-counsel* or tele-diagnos* or tele-health or tele-medic* or tele-monitor* or tele-nursing or telecare or teleconsult* or telecounsel* or telediagnos* or telehealth* or telemedic* or telemonitor* or telenursing or telephone-app* or text-messag* or wearable* or web-portal* or webportal* or whatsapp* or whats-app* or world-wide-web or worldwideweb or www or 7frica7n or twitter or 8frica8nd or x-box or xbox or smart-watch*).ti,ab,kf.
Part 3: defining the CHWs^b^	Community Health Workers/ or (accompagnateur* or accredited-social-health-activist* or asha* or animator* or auxiliary-nurse* or allied-health* or basic-health-worker* or barefoot* or bare-foot* or birth-attendant* or bridge-to-health-team* or care-group* or case-coordinator* or child-health-worker* or community-health-worker* or chw* or close-to-community-provider* or community-agent* or community-aide* or community-based-practitioner* or community-case-management* or community-coordinator* or community-drug-distributor* or community-health-assistant* or community-health-aide* or community-health-agent* or community-health-care-provider* or community-healthcare-provider* or community-health-extension-worker* or community-health-nurse* or community-health-representative* or community-health-surveyor* or community-health-volunteer* or community-healthcare-worker* or community-health-care-worker* or community-healthcare-provider* or community-health-care-provider* or community-health-promoter* or community-health-care-provider* or community-liaison* or community-nutrition-worker* or community-practitioner* or community-resource-person* or community-surveillance-volunteer* or community-volunteer* or community-worker* or care-group* or dame-health-worker* or door-to-door* or extension-service* or extension-officer* or extension-staff* or extension-worker* or family-planning-agent* or family-advocate* or family-support-worker* or family-welfare-assistant* or family-welfare-worker* or field-based* or grassroots* or grass-roots* or hard-to-reach* or health-activist* or health-aide* or health-agent* or health-care-agent* or healthcare-agent* or health-assistant* or health-auxiliar* or health-care-worker* or healthcare-worker* or health-coach* or health-counselor* or health-development-army* or health-distributor* or health-education* or health-extension* or health-nurse* or health-officer* or health-motivator* or health-outreach* or health-out-reach* or health-promoter* or health-promotor* or health-surveillance-assistant* or health-visitor* or health-worker* or health-volunteer* or home-based-care* or home-care* or home-health* or home-service* or home-visit* or iccm* or imci* or intake-specialist* or lady-health-worker* or lay-aide* or lay-attendant* or lay-consultant* or lay-counselor* or lay-health-advisor* or lay-health-worker* or lhw* or lay-visitor* or lay-worker* or lead-mother* or link-worker* or malaria-agent* or child-health-worker* or child-health-worker* or medical-assistant* or midwife* or mid-wife* or midwive* or mid-wive* or mobile-clinic-team* or mother-coordinator* or mother-leader* or navigator* or nutrition-agent* or nutrition-counselor* or outreach-advocate* or outreach-case-manager* or outreach-educator* or outreach-worker* or out-reach-advocate* or out-reach-case-manager* or out-reach-educator* or out-reach-worker* or parent-liaison* or peer-advisor* or peer-counselor* or peer-educator* or peer-health-advisor* or peer-leader* or peer-supporter* or promotora* or rural-health-auxiliar* or support-worker* or surveillance-volunteer* or traditional-birth-attendant* or village-health-volunteer* or village-health-worker* or vhw* or village-drug-kit-manager* or village-health* or village-health-helper* or voluntary-health-worker* or voluntary-worker* or volunteer*).ti,ab,kf.
Part 4: defining context	exp “Africa South of the Sahara”/ or (angol* or benin* or botswan* or burkina-faso* or 11frica11* or cabo-verd* or 11frica11n* or cape-verd* or central-african-republic* or chad* or comoros* or congo* or cote-d-ivoire* or cote-diIvoire* or 11frica11n* or equatorial-guinea* or 11frica11* or eswatini* or 11frica11n* or gabon* or gambia* or ghana or ghanes* or guinea* or ivory-coast* or kenya* or 11frica11* or 11frica11* or madagasca* or 11frica* or mali or malines* or 11frica11nd11* or mauriti* or mozambiq* or 11frica11* or niger or 11frica11* or 11frica11n* or ruand* or rwand* or sao-tome-and-principe* or sao-tome-principe* or 11frica11* or seychell* or sierra-leone* or 11frica11* or south-africa* or rio-muni or 11frica11nd11n-africa* or sub-saharan-africa* or subsahara-africa* or sub-sahara-africa* or sudan* or 11frica11nd* or swazi-land* or tanzan* or togo or togoles* or ugand* or 11frica* or zimbabw* or central-africa* or east-africa* or eastern-africa* or southern-africa* or west-africa* or western-africa* or 11frica-south-of-the-sahara).ti,ab,kf,pl,in.
Part 5: subject combinations	1 and 2 and 3 and 4
Parts 6 and 7: setting predefined criteria	5 not (exp Animals/ not exp Humans/) 6 not case reports/

^a^mHealth: mobile health.

^b^CHW: community health worker.

### Study Selection and Data Extraction

JCFK will retrieve all studies from eligible databases based on the search strategy. CK will export all articles to Covidence computer software [35]. Covidence is a web-based collaboration software that is used to screen studies, perform risk-of-bias assessment and extract data. After the removal of duplicates, CK and HRZ will independently review titles and abstracts to select potential articles that meet the inclusion criteria. Following the identification of potentially eligible studies, CK and HRZ will independently review the studies in full to identify studies that meet the inclusion criteria. Disagreements during the title and abstract review and the full-text screening will be resolved by TvdA. An audit trail regarding the included and excluded studies and studies that required discussion will be maintained. Reasons for study exclusion will include studies conducted outside SSA, mHealth use by cadres of health care workers other than CHWs, and studies reporting outcomes other than those specified in this study.

**Figure 1:**
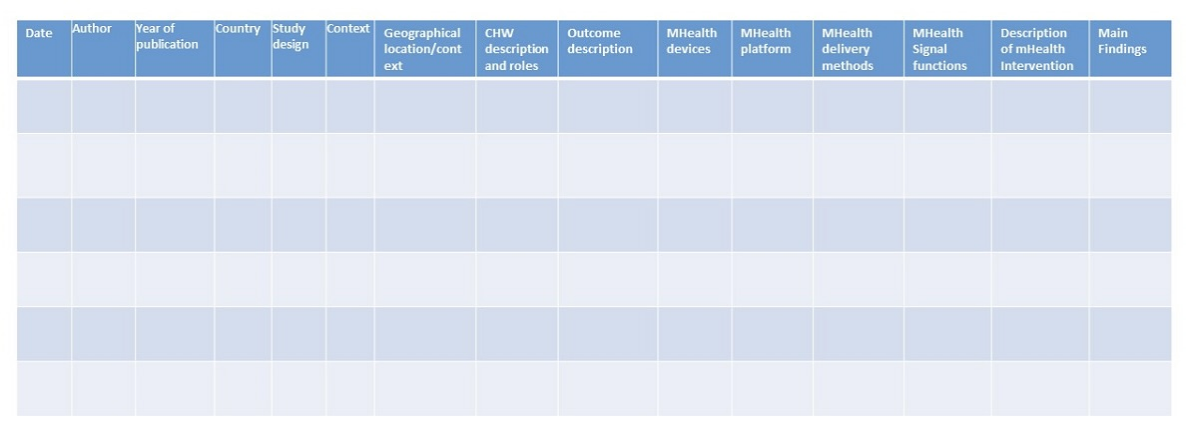
Data extraction tool. CHW: community health workers.

We will design and pretest a data collection tool that will be used in Covidence software (Figure 1). The data collection tool will include the following:

1. Study characteristics: author, year of publication, and study design
2. Context: country and geographical scope
3. Programmatic context: type of CHWs and description of CHW work
4. Mobile health intervention characteristics: mHealth platforms, devices used, delivery method, and intervention description
5. Summary of main findings

### Assessment of Methodological Quality

All included articles will undergo an independent assessment of methodological quality using the Mixed Methods Appraisal Tool (MMAT), 2018 version, by CK and HRZ [36]. MMAT is used to systematically appraise qualitative, quantitative, and mixed methods studies. MMAT is short but comprehensive and easy to use and has been validated and used extensively in reviews [37-40]. Disagreements will be resolved by TvdA. Details of the main domains of the MMAT tool are found in Multimedia Appendix 1. All studies, regardless of the results of their methodological quality, will undergo data extraction and synthesis.

### Data Analysis and Synthesis

**Figure 2:**
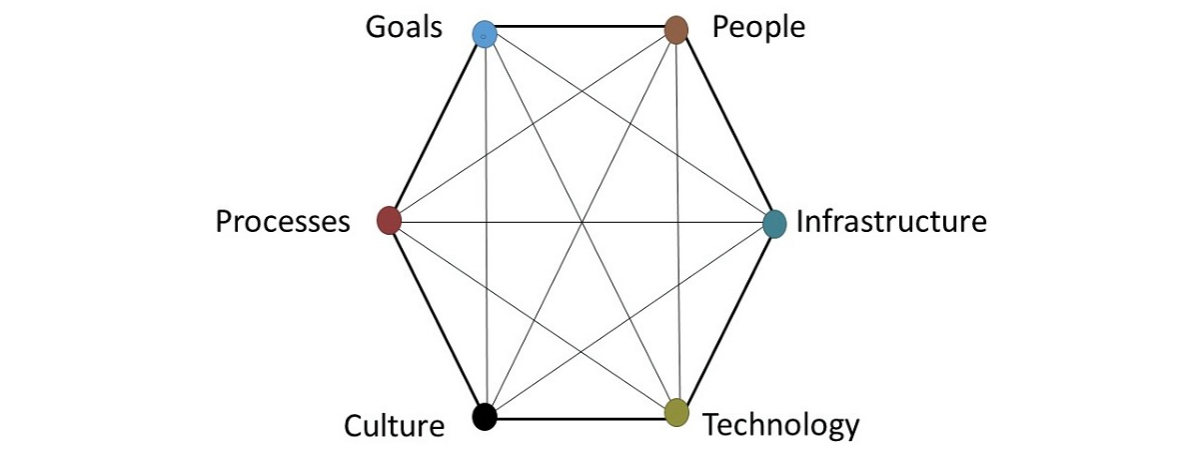
Socio-technical analysis framework.

Since we are including a diverse range of mHealth tools, we anticipate heterogeneous findings from the included studies. As such, we will conduct a narrative synthesis of the results. We will not perform meta-analyses or subgroup analyses. We will follow 3 of the 4 steps of narrative synthesis as outlined by Popay et al [41], which are as follows:

1. A preliminary synthesis of summary findings of the included studies—we will tabulate all results and describe all variables as extracted from eligible studies. For research question 1, we will describe the direction and size of the effect of mHealth across the maternal health continuum of care: ANC, facility-based births, and PNC. For research question 2, we will qualitatively identify barriers and facilitators using a thematic analysis approach [42]. Using this approach, familiarization with the studies will occur during full-text eligibility and data extraction. This will be followed by the development of codes, which will then be organized as themes. The themes will be categorized using the socio-technical analysis framework by Davis et al [43] to group the barriers and facilitators into the following 6 main domains: people, goals, building and infrastructure, technology, culture, and processes and procedures (Figure 2) [43].
2. Exploring the relationships in the data within and between the studies, including explanations of variability in summary findings—we will describe factors that explain the differences in outcomes and barriers and facilitators to mHealth within and between the studies separately. This will be followed by the integration of the results of the two research questions. This is the process in which the two study aims will be linked to describe how mHealth might have led to the effect on maternal health outcomes.
3. A methodological quality assessment for the included studies, as described above.

## Results

In September 2022, we conducted an initial search in the 6 databases plus hand searching in Google Scholar. After removing duplicates, we identified 1111 studies that were eligible for the title and abstract screening. We anticipate finalizing the full-text assessment for eligibility, data extraction, assessment of methodological quality, and narrative synthesis by June 2023. Dissemination of results, including publication of the final manuscript, will follow the PRISMA reporting guidelines [44].

## Discussion

This systematic review will present new and up-to-date evidence on the use of mHealth by CHWs along the pregnancy, childbirth, and PNC continuum of care. Based on the selected databases, we will present a comprehensive synthesis of the existing evidence on the effect of mHealth on the use of maternal health services. Additionally, we will explore the barriers and facilitators of mHealth use, allowing the exploration of factors that shape the implementation of mHealth.

The review is focused on SSA, a region that is currently struggling with the low use rates of maternal health services and identifying ways to improve maternal health outcomes, including a reduction in morbidity and mortality, is a priority [45]. As mHealth use continues to be promoted as one of the innovative ways to improve maternal health use in SSA, we anticipate the results of this review to be important to program implementers, policy makers, and researchers [46,47]. For implementers and policy makers, the results will inform how to use mHealth interventions based on what has been shown to work in other contexts. Additionally, the review will also highlight the present contextual factors that should be addressed to ensure the success of the programs. In the context of implementation, the review has the potential of providing valuable insights on how to modify programs that are currently being implemented to optimize outcomes, support justification for program scale-up, and support the inclusion of mHealth recommendations in policy guidelines. For researchers, we hope that the findings will open a scholarly discussion, create a reproducible process that enables future updates of the review, and based on identified gaps, inform areas of future research. This systematic review is also part of a PhD thesis by the first author.

The review has 2 main limitations. Firstly, we will include peer-reviewed publications only. This may lead to publication bias as some of the information on mHealth may be found in gray literature or may not be published at all. Secondly, we are including maternal health continuum of care outcomes, leaving out other maternal health outcomes.

This systematic review will present the current evidence on the use of mHealth by CHWs in SSA. Therefore, we hope the review will support the use of mHealth for improved maternal health outcomes in SSA.

## Appendix

Multimedia Appendix 1 Details of the main domains of the Mixed Methods Appraisal Tool (MMAT) tool.

## Abbreviations

ANC: antenatal care
CHW: community health worker
mHealth: mobile health
MMAT: Mixed Methods Appraisal Tool
PNC: postnatal care
PRISMA: Preferred Reporting Items for Systematic Reviews and Meta-Analysis
PRISMA-P: Preferred Reporting Items for Systematic Reviews and Meta-Analyses Protocols
SSA: sub-Saharan Africa
